# Cardiac magnetic resonance imaging predictors of atrial arrhythmias in patients with repaired tetralogy of Fallot

**DOI:** 10.1186/1532-429X-17-S1-Q102

**Published:** 2015-02-03

**Authors:** Anne Marie Valente, Shelby Kutty, Matthew White, Jenna Schreier, Jouke Bokma, Edward P Walsh, Michael J Landzberg, Andrew J Powell, Tal Geva

**Affiliations:** 1 Cardiology, Boston Children's Hospital, Boston, MA, USA; 2 Cardiology, Children's Hospital & Medical Center, Omaha, NE, USA

## Background

Patients with repaired tetralogy of Fallot (TOF) experience increased rates of atrial tachyarrhythmias in adulthood. Our objective was to identify cardiac magnetic resonance (CMR) imaging parameters associated with sustained atrial arrhythmias in a large contemporary cohort of patients with repaired TOF.

## Methods

Subjects with repaired TOF with a CMR performed at our institution between 2005-2012 and with clinical follow-up ≥ 1 year or with occurrence of the primary outcome were included. The primary outcome was defined as sustained atrial tachycardia (atrial flutter, atrial fibrillation, or supraventricular tachycardia undergoing arrhythmia-directed therapy), occurring after the index CMR.

## Results

The cohort includes 365 subjects (median age 18.6 years), and consisted of TOF/ pulmonary stenosis (74%), TOF/pulmonary atresia (23%), and TOF/atrioventricular canal (3%). Over 50% of the subjects had undergone a transannular patch repair and 23% had undergone a palliative shunt procedure prior to complete repair. Median age of repair was 0.6 years (0.01 - 45.3 years). Of the 365 subjects, 23 (6%) reached the primary outcome (median age at outcome 30.7 years; median time from CMR to outcome 2.2 years). Univariate Cox proportional hazard regression models identified lower right atrial (RA) fractional area change (for a decrease of 10%, HR=1.86; 95% CI 1.14-3.03; p = 0.013), maximal RA volume index (for an increase of 5ml/m^2^, HR=1.10; 95% CI, 1.04- 1.17; P = 0.002), larger right ventricular (RV) end-diastolic volume (for a 3 standard deviation increase, HR=1.42; 95% CI, 1.04-1.94; P = 0.029), and less pulmonary regurgitation (PR) (for a 5% decrease, HR=1.14; 95% CI, 1.01-1.28; p = 0.03) as outcome predictors (Table 1). The degree of tricuspid regurgitation was not associated with the outcome.

**Table 1:** Predictors of Atrial Arrhythmias in Patients with Repaired TOF

CMR Variables		HR	95% CI	p value	C Index
Max RA volume index (mL/m^2^)	↑5	1.10	(1.04, 1.17)	0.002	0.604

Min RA volume index (mL/m^2^)	↑5	1.13	(1.06, 1.21)	<0.001	0.619

RA FAC (%)	↓10	1.86	(1.14, 3.03)	0.013	0.615

RA ejection fraction (%)	↓10	1.37	(1.01, 1.88)	0.046	0.596

RV diastolic volume z-score	↑3	1.42	(1.04, 1.94)	0.029	0.585

RV systolic volume z-score	↑3	1.25	(1.06, 1.48)	0.007	0.564

RV ejection fraction (%)	↓10	1.29	(0.88, 1.89)	0.19	0.446

RV mass z-score	↑3	1.31	(0.84, 2.04)	0.24	0.474

Tricuspid regurgitation (%)	↑5	1.11	(0.86, 1.44)	0.43	0.517

Pulmonary regurgitation (%)	↓5	1.14	(1.01, 1.28)	0.03	0.612

LV diastolic volume z-score	↑3	1.54	(0.96, 2.48)	0.07	0.621

LV systolic volume z-score	↑3	1.21	(0.9, 1.65)	0.21	0.593

LV ejection fraction (%)	↓10	1.16	(0.71, 1.89)	0.56	0.510

LV mass z-score	↑3	1.45	(0.97, 2.16)	0.07	0.581

FAC, fractional area change; HR, hazard ratio; LV, left ventricle; RA, right atrial; RV, right ventricle

## Conclusions

Larger RA and RV size and lower RA function are predictive of atrial tachyarrhythmias in adults with repaired TOF. Given these findings, as well as a lower degree of PR in patients with atrial tachyarrhythmias suggests that a decrease in RV compliance may play a role in the outcome.

## Funding

Higgins Family Noninvasive Research Fund at Boston Children's Hospital; The Lerner Research Award at Brigham and Women's Hospital.

